# Symbiotic essential amino acids provisioning in the American cockroach, *Periplaneta americana* (Linnaeus) under various dietary conditions

**DOI:** 10.7717/peerj.2046

**Published:** 2016-05-18

**Authors:** Paul A. Ayayee, Thomas Larsen, Zakee Sabree

**Affiliations:** 1 Department of Evolution, Ecology and Organismal Biology, Ohio State University, USA; 2 Laboratory for Radiometric Dating and Stable Isotope Research, Christian-Albrechts-Universität Kiel, Kiel, Germany

**Keywords:** Essential amino acids, Insect host, δ^13^C_EAA_ analyses, Gut microbes, Periplaneta americana, Symbiotic EAA provisioning

## Abstract

Insect gut microbes have been shown to provide nutrients such as essential amino acids (EAAs) to their hosts. How this symbiotic nutrient provisioning tracks with the host’s demand is not well understood. In this study, we investigated microbial essential amino acid (EAA) provisioning in omnivorous American cockroaches (*Periplaneta americana)*, fed low-quality (LQD) and comparatively higher-quality dog food (DF) diets using carbon stable isotope ratios of EAAs (*δ*^13^C_EAA_). We assessed non-dietary EAA input, quantified as isotopic offsets (Δ^13^C) between cockroach (*δ*^13^C_Cockroach EAA_) and dietary (*δ*^13^C_Dietary EAA_) EAAs, and subsequently determined biosynthetic origins of non-dietary EAAs in cockroaches using ^13^C-fingerprinting with dietary and representative bacterial and fungal *δ*^13^C_EAA_. Investigation of biosynthetic origins of *de novo* non-dietary EAAs indicated bacterial origins of EAA in cockroach appendage samples, and a mixture of fungal and bacterial EAA origins in gut filtrate samples for both LQD and DF-fed groups. We attribute the bacteria-derived EAAs in cockroach appendages to provisioning by the fat body residing obligate endosymbiont, *Blattabacterium* and gut-residing bacteria. The mixed signatures of gut filtrate samples are attributed to the presence of unassimilated dietary, as well as gut microbial (bacterial and fungal) EAAs. This study highlights the potential impacts of dietary quality on symbiotic EAA provisioning and the need for further studies investigating the interplay between host EAA demands, host dietary quality and symbiotic EAA provisioning in response to dietary sufficiency or deficiency.

## Introduction

Insects persisting on diets limited in essential nutrients are posited to rely on mutualistic symbiosis (obligate or facultative) with microbes to acquire these nutrients and meet requirements for growth, fecundity, longevity and ultimately, fitness (Douglas, 2009). Evidence of obligate endosymbiont nutrient provisioning and the influence of this on host fitness has been demonstrated in several insect-obligate endosymbiont systems, such as the pea aphid-*Buchnera* (Prosser & Douglas, 1991; Douglas, Minto & Wilkinson, 2001; Russell et al., 2014), and tsetse fly-*Wigglesworthia* (Pais et al., 2008). However, microbial species diversity and the complexity of interspecies interactions in the guts of insect hosts make ascertaining the contributions and functions of gut-associated microbiota challenging. This difficulty is further compounded by considerable variations in community composition with time and host developmental stages, as well as differences in physicochemical requirements (oxygen and pH) needed by community members in order to perform particular functions (Engel & Moran, 2013).

The American cockroach (*Periplaneta americana*), is a widely distributed omnivore that thrives on decaying plant and animal materials and occasionally conspecific carcasses (Bell, Roth & Nalepa, 2007). Nearly all cockroaches have the obligate intracellular bacterial symbiont *Blattabacterium* sp., located in their fat bodies (Sabree, Kambhampati & Moran, 2009). Analyses of various *Blattabacterium* genomes suggests that nitrogen recycling and essential amino acid (EAA) provisioning are vital functions in these associations (Sabree, Kambhampati & Moran, 2009). Cockroaches additionally have gut microbiota that are similar in composition to that of termites, a closely related group of insects (Sabree & Moran, 2014; Schauer, Thompson & Brune, 2012). Biosynthetic and degradative functions such as cellulose degradation (Brune, 2014; Scharf et al., 2011), nitrogen fixation (Lilburn et al., 2001) and reductive acetogenesis (Brune, 2014) by termite gut microbiota, have been similarly proposed for cockroach gut microbiota based on the presence of shared functionally relevant bacterial taxa (Schauer, Thompson & Brune, 2012; Sabree & Moran, 2014). The impacts of dietary quality on cockroach gut microbial community composition has been shown to be context-dependent. For example, Firmicutes are more abundant in cockroaches fed crystalline cellulose, relative to Bacteroidetes, Proteobacteria and Synergistetes in wild-caught and sugarcane bagasse-fed cockroaches (Bertino-Grimaldi et al., 2013). On the other hand, no difference in community composition was detected in cockroaches fed a high or low fiber diet (Schauer, Thompson & Brune, 2014). The implications of these diet-induced effects on microbiota composition and functions, such as EAA provisioning remain unclear.

In this study, we investigated microbial EAA provisioning in *Periplaneta americana* fed diets varying in protein contents. To investigate EAA provisioning we used carbon stable isotope values of essential amino acids (*δ*^13^C_EAA_) basing our analysis on two premises. First, cockroaches are incapable of *de novo* EAA synthesis and therefore rely on dietary or non-dietary/symbiotic sources for these. If EAAs derive strictly from the diet, EAA carbon isotope values of cockroaches (*δ*^13^C_Cockroach EAA_) fed a particular diet are expected to match those of the diet (*δ*^13^C_Dietary EAA_) (McMahon et al., 2010; Newsome et al., 2011). This means EAAs are taken up from diets with little to no chemical alterations, and consequently no significant differences between mean cockroach (*δ*^13^C_Cockroach EAA_) and dietary (*δ*^13^C_Dietary EAA_) carbon isotope EAAs values are anticipated. Significant differences between cockroaches and dietary carbon isotope values are therefore suggestive of potential symbiotic EAA provisioning (Newsome et al., 2011). This significant difference can then be expressed/visualized as isotopic offsets (Δ^13^C) between cockroach and dietary *δ*^13^C_EAA_ (${\Delta }^{13}\mathrm{C}={\delta }^{13}{\mathrm{C}}_{\mathrm{Cockroach}~\mathrm{EAA}}-{\delta }^{13}{\mathrm{C}}_{\mathrm{Dietary}~\mathrm{EAA}}$). A prerequisite of this premise is that, the diet(s) of the insect/consumer is known. The choice of diet is predetermined in controlled laboratory settings, such as this study, but is important to establish this for field studies.

The second premise is that bacteria and fungi generate distinct *δ*^13^C_EAA_ signatures (Larsen et al., 2009). It is therefore possible to determine whether the non-dietary/symbiotic EAA input is of bacterial or fungal origin. This is carried out within a predictive model framework using microbial (bacterial and fungal) and dietary *δ*^13^C_EAA_ as classifiers to determine group membership of cockroach *δ*^13^C_EAA_. This approach, called ^13^C-fingerprinting technique has been used to determine biosynthetic origins of EAAs in several systems (Larsen et al., 2009; Larsen et al., 2011; Larsen et al., 2013; Vokhshoori, McCarthy & Larsen, 2014; Ayayee, Jones & Sabree, 2015; Ayayee et al., 2015). We hypothesized that microbial EAA provisioning will be greater in cockroaches fed a low protein diet relative to those fed a high protein diet. This would be illustrated as greater isotopic offsets between cockroaches fed a low protein diet and their diet, as well as greater number of low protein diet fed cockroaches assigned to microbial (bacteria or fungi) classifiers in the predictive model.

## Methods and Materials

### Source of insects

The 5th instar *P. americana* nymphs used in this study were obtained from a colony maintained in the insectary of the Department of Evolution, Ecology and Organismal Biology, at the Ohio State University, Columbus, Ohio. Considerable difficulties associated with confidently eliminating *Blattabacterium* from cockroach fat bodies necessitated the decision to use *Blattabacterium*-infected cockroaches of convenience and availability. Prior to experimentation, insects were maintained in a growth chamber (27 °C and 29% relative humidity) and fed dog food pellets (27% crude protein).

### Dietary experiments

Individual 5th instar *P. americana* nymphs were placed on diets varying in protein contents. We qualified the low protein and assimilable carbon diet as the low-quality diet (LQD). This was composed of 10 g basal protein mix and 50 g cellulose. The basal protein mix (55% protein) made up the defined protein source, and consisted of: yeast extract (10 g), Hawk-Oser # 3 salt mix (4 g), casein (45 g), and dextrin (41 g). Combining 10 g of the basal protein mix with 50 g of cellulose resulted in a final protein content of 9.16% and cellulose content of 83.3% in the low-quality diet (LQD). Dog food (DF) (Red Flannel™ Hi-Protein Formula; PMI Nutrition, St. Louis, MO, USA) was qualified as the high protein diet. Dog food has been used to rear cockroaches in several laboratories, ours included, and has not been shown to negatively impact *P. americana* growth and longevity. The DF diet consisted of: crude protein 27%, crude fiber 4%, calcium 1%, zinc 1,225 ppm, vitamin E 80 IU/kg, crude fat 12%, moisture 12%, phosphorus 0.80%, vitamin A 10,000 IU/kg and omega-6-fatty acids 1.5%. All nymphs were reared individually at room temperature and relative humidity. Likely sources of proteins in the crude protein fraction are meat and bone meal, soybean meal and brewer’s dried yeast. The food was changed weekly and water provided *ad libitum* over a period of 8 weeks, at which point high mortality among the LQD-fed cockroaches forced us to terminate the study. Nymphs used in the LQD dietary group were reared on dog food prior to switching diet because we had concerns regarding nymph mortality and longevity, if fed LQD upon hatching until the experiment started.

### Sample preparation

At the end of the feeding period, three LQD-fed cockroaches (*n* = 3) and four DF-fed cockroaches (*n* = 4) were surface-sterilized by rinsing once in 20 ml 10× dilution (10 ml concentrated detergent: 90 ml Milli-Q water) of detergent solution (Coverage Plus; Steris, Mentor, OH, USA) and twice in sterile Milli-Q water. The entire alimentary system from each cockroach was removed and homogenized in 5 ml of 1× dilution of phosphate buffer solution (PBS) (100 ml of 10× concentrate PBS: 900 ml Milli-Q water). Homogenates containing putative unassimilated microbial EAAs, were filtered through a 0.45 µm membrane filter (EMD Millipore, Billerica, MA, USA) to exclude insect debris and filtrate collected into a 1.5 ml collection tube (Eppendorf, Hauppauge, NY, USA). Six appendages, representative of insect assimilated EAAs were also collected from each of these eviscerated individuals. All samples were stored at −80 °C for 48 h prior to lyophilization and pulverized after lyophilization. Two technical replicates of all insect appendage and gut filtrate samples and three technical replicates of the LDQ and DF diets were collected into 1.5 ml collection tubes, and submitted for ^13^C-stable isotope analysis at the Stable Isotope Facility (SIF) at UC Davis (Davis, California, USA). Thus for each cockroach we acquired filtered gut homogenate, as well as appendage samples.

### *δ*^13^C_EAA_ quantification and analysis

All samples were acid hydrolyzed and derivatized in a solution of methanol, pyridine and methyl chloroformate using a one-step rapid derivatization method (Walsh, He & Yarnes, 2014; Chen et al., 2010). Approximately 0.35 µl aliquots of derivatized samples were then injected into a splitless liner at 250 °C with a Helium flow rate of 2.8 mL/min. Conditions and optimization during derivatization and analysis were performed as previously reported (Chen et al., 2010; Walsh, He & Yarnes, 2014). Analyses were carried out using a Trace gas chromatograph (Thermo Fisher Scientific) coupled to a Delta V Advantage isotope ratio mass spectrometer via the GC Combustion Interface III (Thermo Electron, Bremen, Germany) using the high polar VF-23ms column (Agilent Technologies). Combustion and reduction furnace temperatures were 950 °C and 650 °C, respectively.

*δ*^13^C_EAA_, defined as $[({R}_{\mathrm{sample}\hspace*{1em}\mathrm{EAA}}/{R}_{\mathrm{standard}\hspace*{1em}\mathrm{EAA}})-1]\times 1\text{,}000$‰, where *R* is the ratio of heavy to light isotope in EAA of the sample, *R*_sample EAA_, and standard, *R*_standard EAA_, was determined for each sample and calibrated to the international *δ*^13^C standard, Vienna Pee Dee Belemnite (V-PDB) scale (Coleman & Fry, 1991). Two technical replicates per biological sample were analyzed. Correction for the addition of carbon during derivatization was performed after analysis (Walsh, He & Yarnes, 2014; Chen et al., 2010). Distinct peaks without overlaps were obtained for the EAAs isoleucine, leucine, valine, phenylalanine and lysine from the capillary column of the gas chromatograph for all samples. The carbon-corrected *δ*^13^C_EAA_ values of leucine (Leu), isoleucine (Ile), lysine (Lys), phenylalanine (Phe), and valine (Val), were obtained.

### Statistical analyses

Mixed model analysis and mean separations (Student’s *t*-test, *P* = 0.05) were carried out for the LQD and DF *δ*^13^C_EAA_ data using JMP 10 (SAS Inc. NC, USA). Individual Δ^13^C-offsets between the 5 EAAs for cockroach and dietary samples were determined as: ${\Delta }^{13}{\mathrm{C}}_{\mathrm{EAA}}={\delta }^{13}{{\mathrm{C}}_{\mathrm{Cockroach}}}_{\mathrm{EAA}}-{\delta }^{13}{\mathrm{C}}_{\mathrm{Diet}\hspace*{1em}\mathrm{EAA}}$. The groups were; low-quality diet (LQD), LQD-fed roach appendages, LQD-fed roach gut filtrate, dog food (DF), DF-fed roach appendages and DF-fed roach gut filtrate.

We utilized a linear discriminant function analysis (LDA) using *δ*^13^C_EAA_ training data from reference bacterial and fungal samples (Larsen et al., 2013), and the LQD and DF dietary samples to generate the predictive model. The *δ*^13^C_EAA_ data obtained from the fungus *Fusarium solani* (*n* = 2), used in a previous study and analyzed from the same facility as these samples, was included in the model for validation (Ayayee et al., 2015). Classification of known fungus sample and group membership of cockroach samples in relation to the classifiers was determined using the R package MASS (Venables & Ripley, 2002).

## Results

### Δ^13^C-offsets detected between LQD-fed and DF-fed cockroaches and respective diets

**Figure 1:**
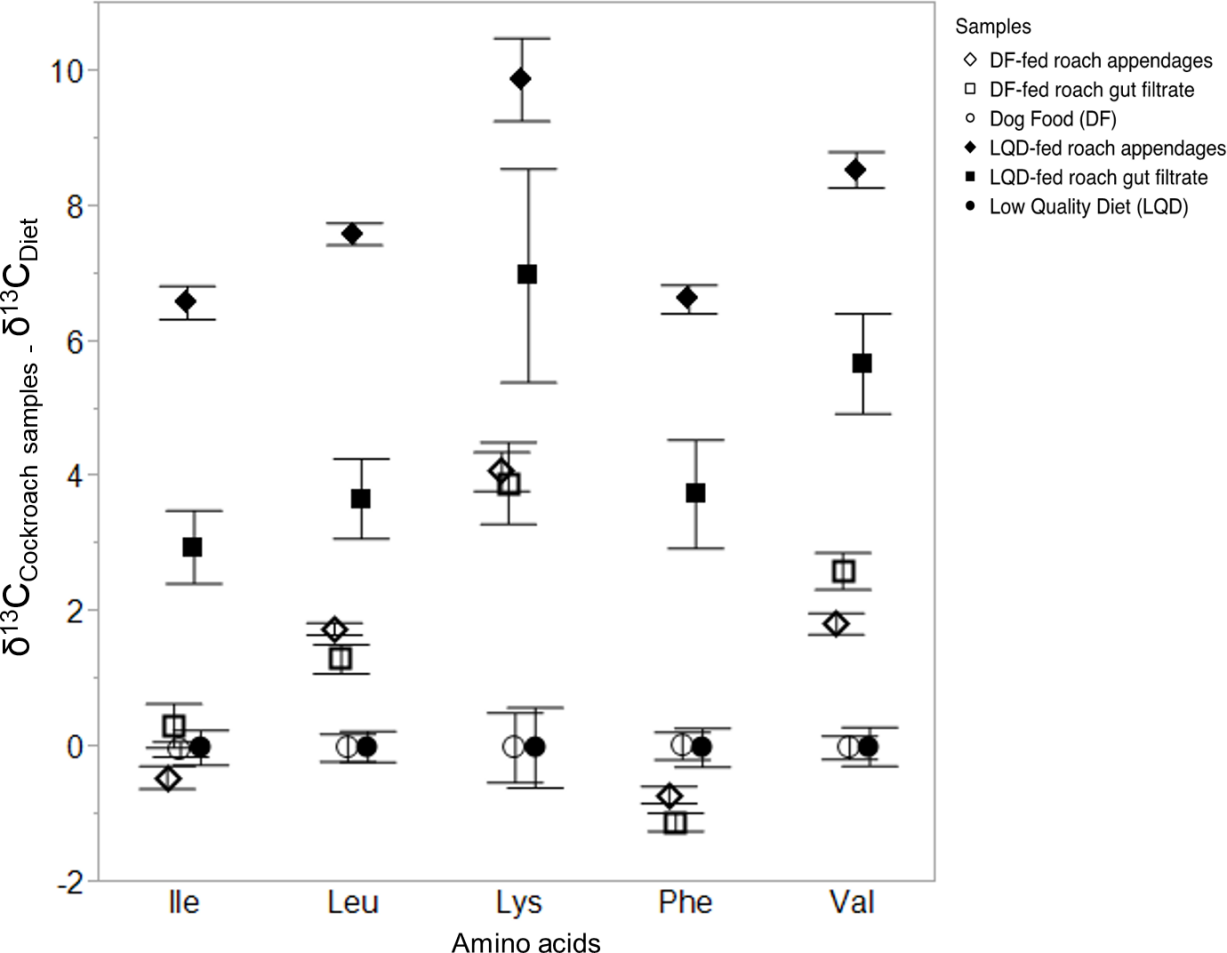
Isotopic offsets (${\Delta }^{13}\mathrm{C}={\delta }^{13}{\mathrm{C}}_{\mathrm{Cockroach}\hspace*{1em}\mathrm{EAA}}-{\delta }^{13}{\mathrm{C}}_{\mathrm{Diet}\hspace*{1em}\mathrm{EAA}}$) (mean ± s.e.) between DF-fed and LQD-fed roach samples (appendages and gut filtrates) normalized to the DF and LQD diet respectively, determined for five essential amino acids. The EAAs used were isoleucine (ile), leucine (leu), lysine (lys), phenylalanine (phe), and valine (val). Shown are offsets for the LQD-fed roach appendage, LQD-fed roach gut filtrate, and the LQ diet (*n* = 3, each), as well as the DF diet (*n* = 3), DF-fed roach appendage and DF-fed roach gut filtrate samples (*n* = 4, each).

A significant overall model effect was determined for both the DF-fed and the LQD-fed cockroach samples and their respective diets (*F*_(29,160)_ = 110, *P* < 0.0001). Significant differences in mean *δ*^13^C_EAA_ were detected among all groups across all 5 EAAs measured (*F*_(5,184)_ = 263, *P* < 0.0001), with both LQD and DF-fed roach appendage and gut filtrate samples significantly different from the DF and LQD diets, as well as from each other (Table 1). Mean positive ${\Delta }^{\mathbf{13}}$C-offset for the DF-fed roach appendage and DF-fed gut filtrate samples were respectively, 1.30 ± 0.18‰ and 1.4 ± 0.18‰ (mean ± s.e.) relative to the DF-diet (0‰), whereas mean Δ^13^C-offset between the LQD-fed roach appendages and LQD-fed roach gut filtrate samples were respectively 7.8 ± 0.2‰ and 4.6 ± 0.2‰ (mean ± s.e.), relative to the LQD diet (0‰) (Table 1). Significant differences in the *δ*^13^C_EAA_ of the 5 EAAs measured were also determined (*F*_(4,185)_ = 411, *P* < 0.0001). The Δ^13^C-offsets between the DF-fed and LQD-fed cockroach samples and their respective diets for each of the 5 EAAs are shown in Fig. 1. For both the DF-fed and LQD-fed cockroach samples, offsets were greatest for lysine and valine (i.e., ^13^C-enriched), followed by leucine, isoleucine and phenylalanine. Overall, degree of Δ^13^C-isotopic offset was higher in the LQD-fed cockroach samples (6.2 ± 0.2‰, mean ± s.e.), compared to the DF-fed cockroach samples (1.35 ± 0.18‰, mean ± s.e.) (Fig. 1). The *δ*^13^C_EAA_ data used in the analyses are presented in Table S1.

**Table 1:** Mean *δ*^13^C_EAA_ (± s.e.) and calculated isotopic offset (Δ^13^C_EAA_) for both LQD-fed and DF-fed cockroach samples (gut filtrate and appendages), and respective LQ and DF diets (ANOVA: *F*_(5,185)_ = 263, *P* < 0.0001). Significantly different samples are indicated by different letters following the Student’s *t*-test at *P* = 0.05. Different standard errors are due to different sample sizes.

Samples and replicates	Mean *δ*^13^C_EAA_ (per mil) ± S.E.	Isotopic offsets (Δ^13^C_EAA_)
DF-fed roach gut filtrate (*n* = 4)	−20.9 ± 0.2 (A)	1.4
DF-fed roach appendage (*n* = 4)	−21.0 ± 0.2 (A)	1.3
Dog food diet (*n* = 2)	−22.0 ± 0.3 (C)	0
LQD-fed roach gut filtrate (*n* = 3)	−24.9 ± 0.2 (D)	4.6
LQD-fed roach appendage (*n* = 3)	−21.6 ± 0.2 (B)	7.8
Low-quality diet (LQD) (*n* = 3)	−29.5 ± 0.2 (E)	0

### Predictive model description and validation

LDA was used to determine group membership of cockroach samples to either the bacterial, fungal or dietary classifier groups. In the LDA plots, the 95% confidence limits for classifier groups are depicted as ellipses (dashed lines) and the decision boundaries between the classifier groups are depicted as dotted lines separating the classifiers. Posterior probabilities, i.e., the probability that a particular sample belonged to one or another of the three classifier groups were then predicted following model establishment. The greater the distance of a particular consumer from the centroid of a classification group (i.e., potential EAA source) the greater the probability mixing of EAA sources occurred. Discriminant scores of consumers falling outside the 95% confidence limits of their dietary sources are interpreted as strong indicators of non-dietary/symbiotic EAA provisioning given the distinct discrimination scores between the classifiers.

**Table 2:** Summary of the predictive model based on classification and posterior probability scores of the fungal (*n* = 7) and bacterial (*n* = 12) classifiers, and the LQD (*n* = 3) and DF (*n* = 2) used in the training dataset in the LDA analysis.

	Probability (%)		
Actual sample	Bacteria	Diet	Fungi
Fungi	0.00	18.40	81.60
Fungi	0.00	0.54	99.46
Fungi	0.00	0.26	99.74
Fungi	0.00	0.00	100.00
Fungi	0.00	0.00	100.00
Fungi	0.00	0.38	99.62
Fungi	0.00	0.07	99.93
Bacteria	100.00	0.00	0.00
Bacteria	100.00	0.00	0.00
Bacteria	100.00	0.00	0.00
Bacteria	100.00	0.00	0.00
Bacteria	100.00	0.00	0.00
Bacteria	100.00	0.00	0.00
Bacteria	100.00	0.00	0.00
Bacteria	100.00	0.00	0.00
Bacteria	100.00	0.00	0.00
Bacteria	100.00	0.00	0.00
Bacteria	100.00	0.00	0.00
Bacteria	99.99	0.01	0.00
Low-quality diet (LQD)	0.00	99.99	0.01
Low-quality diet (LQD)	0.00	99.99	0.01
Low-quality diet (LQD)	0.00	100.00	0.00
Dog food diet	0.00	96.36	3.64
Dog food diet	0.00	97.49	2.51

**Figure 2:**
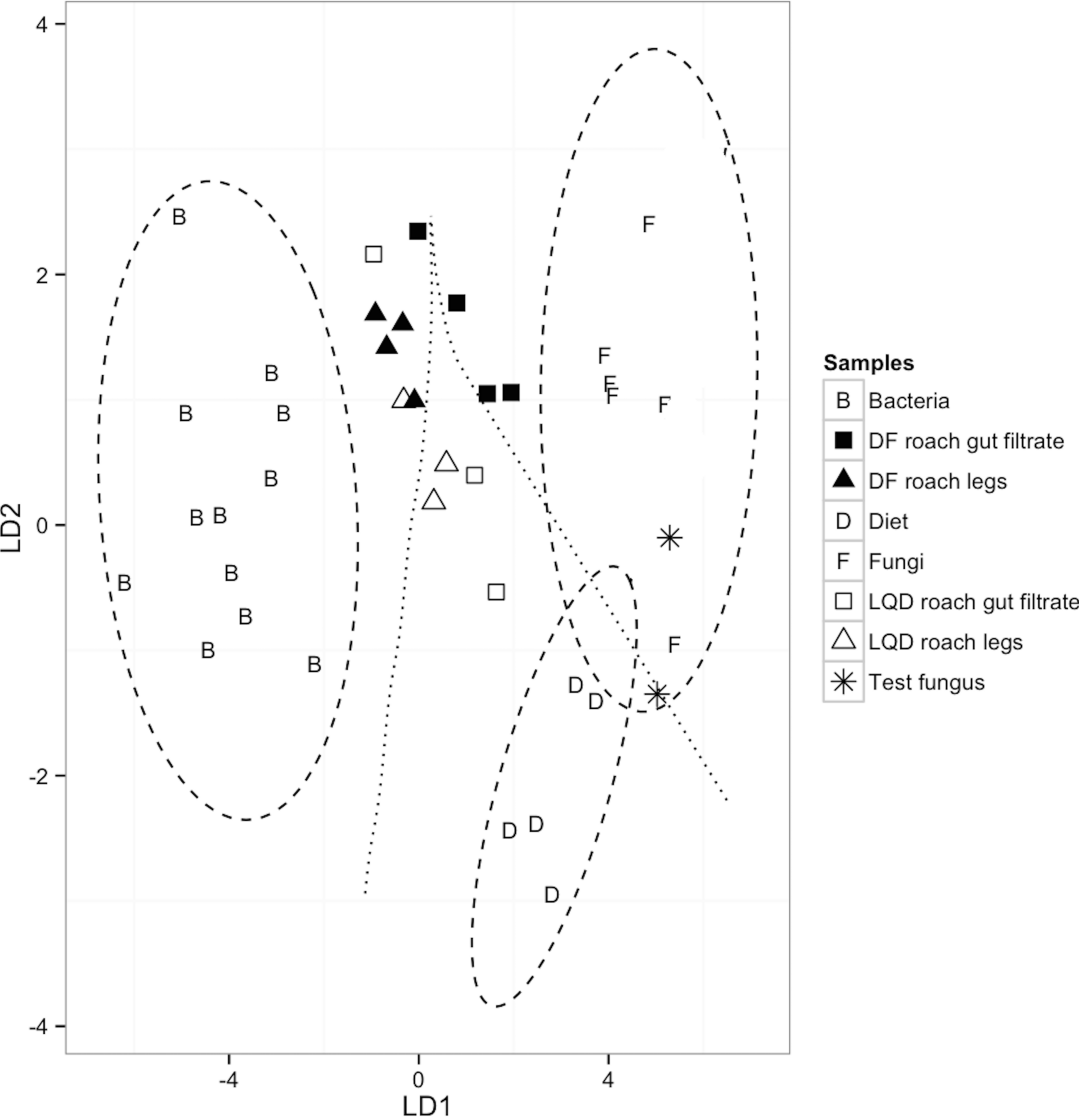
A linear discriminant analysis (LDA) plot showing group assignments of LQD-fed and DF-fed cockroach samples to classifiers; Diet (*n* = 5; LQD diet, 3 and DF diet, 2), fungi (*n* = 7), bacteria (*n* = 12) and (*F*_(10,34)_ = 20.13, *P* < 0.0001; Wilk’s lambda = 0.020; LD1 = 92.6%, LD2 = 7.4%). The 95% confidence limits decision regions for each group/classifier are depicted as ellipses around the classifiers and the decision boundaries between the groups/classifiers as lines. Cockroach samples outside the 95% confidence limit decision region represent samples with non-dietary EAA input. The two dietary samples closest to the fungal classifier are the DF diets. The EAAs used were: isoleucine (ile), leucine (leu), lysine (lys), phenylalanine (phe), and valine (val).

Validation of the model was confirmed via the correct and distinct separation of bacterial (*n* = 12) and fungal (*n* = 7) samples, in the training dataset, to their respective groups (*F*_(15,78)_ = 12.6, *P* < 0.0001; Wilk’s lambda = 0.03, a test of appropriateness of classifiers in predicting group membership of predictors) (Table 2 and Fig. 2). The model also classified the dietary sources (LQ diet, *n* = 3 and DF diet, *n* = 2), as distinct from bacterial classifier group, but similar to the fungal classifier group. This most likely reflects contribution of the fungal proteins (yeast extract) in the LQ diet, and the fungal component (brewer’s yeast) of the crude protein fraction of the DF diet. Finally, the validity of the model was further confirmed by the correct placement of the two *F. solani* fungal samples, within the 95% confidence limit of the fungal classifier group (Fig. 2).

### Classification of cockroach samples by the model

None of the cockroach appendage and gut filtrate samples had discriminant scores within the 95% confidence limit decision region of respective dietary sources. Based on the placement of the cockroach samples in the LDA plots, bacteria and fungi appear equally likely to be sources of non-dietary EAA input in the LQD-fed cockroach samples. Two out of the three LQD-fed cockroach appendage and gut filtrate samples were within the decision boundary of the dietary classifiers, but positioned towards the bacterial classifier group (Fig. 2). The two LQD-fed cockroach appendage samples (open triangles) were positioned closer to the bacterial classifier than the LQD-roach gut filtrate samples (open squares) (Fig. 2). The remaining LQD-fed cockroach appendage and gut filtrate samples (one each) were located within the decision boundary of the bacterial classifier group, suggestive of bacterial EAA input (Fig. 2). All four DF-fed cockroach appendage samples and one DF-fed cockroach gut filtrate sample were located within the decision boundary of the bacterial classifier group, suggestive of bacterial EAA input (Fig. 2). The remaining three DF-fed cockroach gut were placed within the fungal decision boundary.

## Discussion

In this study, we uncovered symbiotic (gut microbial and *Blattabacterium*) EAA inputs in both LQD-fed and DF-fed *P. americana* cockroaches under controlled-feeding conditions using *δ*^13^C_EAA_ analyses, although EAA provisioning was comparatively higher in the DF-fed cockroaches, contrary to our hypothesis. We make no distinction between EAA provisioning by gut microbes or the fat body-residing obligate symbiont *Blattabacterium* since we did not use *Blattabacterium*-free cockroaches. Aspects of symbiotic EAA provisioning under both dietary conditions, and the potential sources of non-dietary EAAs are discussed below.

Gut residing microbes have been demonstrated to serve as sources of non-dietary EAAs in insects such as the eastern subterranean termite *Reticulitermes flavipes* (Ayayee, Jones & Sabree, 2015), and the Asian long horned beetle *Anoplophora glabripennis* (Ayayee et al., 2015), which do not have obligate endosymbionts in the strictest sense. Although the possibility has been suggested that there might be fat body residing bacteria associated with some long horned beetles (Calderon & Berkov, 2012), this remains to be definitively determined. One interpretation of the results from this study is that the observed symbiotic EAA input detected in the cockroach samples derives from gut microbial EAA provisioning. The exact mechanisms by which gut microbe-derived EAAs are made available and taken up by the cockroach host are unclear. Possible routes include digestion, lysis and uptake of EAA from gut microbial residents by the insect host (Douglas, 2009) and the acquisition of microbial EAAs through coprophagy i.e., the re-ingestion of fecal materials containing partially digested food debris and microbial cells (Nalepa, Bignell & Bandi, 2001; Zimmer & Topp, 2002; Bell, Roth & Nalepa, 2007). Higher dietary quality in the DF diet may be accompanied by greater bacterial densities in DF-fed cockroach guts, which may be responsible for the higher EAA input. However, gut microbial EAA provisioning using *Blattabacterium*-free cockroaches, followed by quantification of absolute bacterial loads under different dietary conditions need to be investigated in order for this to be ascertained.

The contributions of the obligate endosymbiont *Blattabacterium* (Strain BPLAN) factors significantly in the discussion of EAA provisioning in cockroaches, since *Blattabacterium* can synthesize all of the five quantified EAAs in this study, as well as recycle nitrogen (Sabree, Kambhampati & Moran, 2009). The determination of symbiotic EAA input in both the DF and LQD-fed cockroaches in this study can therefore be interpreted as indicative of *Blattabacterium* EAA input. In the LQD-fed and DF-fed cockroaches used in this study, *Blattabacterium*-derived EAAs are most likely transported out from bacteriocytes via amino acid transporters and assimilated directly by the host in the fat body under both dietary conditions (Sabree, Kambhampati & Moran, 2009). In a taxonomically unrelated system, EAA provisioning by the obligate endosymbiont of pea aphids, *Buchnera,* has been proposed to be regulated by the flux of precursors metabolites available for EAA biosynthesis (Reymond et al., 2006; Russell et al., 2014). Precursor metabolite availability can be potentially influenced by dietary quality. Cockroach gut microbial members have been shown to be capable of synergistic lignocellulose degradation (Bertino-Grimaldi et al., 2013), presumably providing intermediate products such as glucose, acetate, etc., to the host, which can subsequently be routed to *Blattabacterium* for EAA biosynthesis. In the LQ diet-fed cockroaches, it is likely that recalcitrant pure cellulose in the LQ diet (protein content, 9.16%, cellulose content, 83.3%) slowed down the flux of precursor metabolites to *Blattabacterium*, by impacting the presence and abundances of lignocellulolytic bacteria, as well as other bacteria, subsequently increasing the time it takes for pure cellulose to get degraded synergistically and the amount of metabolites routed to *Blattabacterium*. This remains to be investigated further in this species. Conversely, the flux of precursor metabolites to *Blattabacterium* for EAA biosynthesis might be higher in the DF-fed cockroaches than in LQD-fed cockroaches, as a result of the higher quality of the diet, resulting in the observed higher EAA input.

Symbiotic EAA provisioning uncovered in this study however, need not be exclusively *Blattabacterium* or gut microbial, and is likely a function of both. We believe the interpretation of the results from this study as indicative of symbiotic EAA input (*Blattabacterium* and gut microbial) in both the DF-fed and LQD-fed cockroaches has it merits, despite the limited sample sizes. We sought to utilize the *δ*^13^C_EAA_ approach to provide data to bridge the gap between potential symbiont capabilities, such as EAA provisioning (evidenced from genomic, metagenomic and metatranscriptomics studies), and demonstrated symbiont function, such as EAA transfer between symbiont and host (based on ^13^C-fingerprinting analyses). This study represents the first investigation of symbiotic EAA provisioning in *P. americana* using this technique and provides the basis for further studies aimed at disentangling gut microbial and *Blattabacterium* EAA input using *Blattabacterium*-free and infected individuals under different dietary conditions.

The comparatively greater symbiotic EAA provisioning observed in the DF-fed cockroach samples (based on the number of DF-fed cockroach appendage samples within the bacterial decision boundary by the predictive model) relative to LQD-fed roach samples was unexpected. This however, may be attributed to the factors outlined above. The lower than anticipated symbiotic EAA input in the LQD-fed roach samples despite the greater isotopic offsets was similarly unexpected. It can be argued that the observed EAA isotopic offsets between LQD-fed roaches and the LQ diet are due to a combination of low EAA turnover rates (O’Brien, Fogel & Boggs, 2002; Martinez del Rio et al., 2009) and the truncated feeding period, as opposed to actual non-dietary EAA input, since the LQD-fed cockroaches were fed the DF diet from hatching till the start of the experiment. However, this is unlikely given the differences in EAA Δ^13^C-offsets between DF-fed and LQD-fed roach samples (Fig. 1), as well as the overall differences in mean *δ*^13^C_EAA_ of DF-fed and LQD-fed roach samples (Table 1), indicative of isotopic equilibration of LQD-fed roaches with the LQ diet. Thus, *de novo* symbiotic EAA input evidenced by the positioning of LQD-fed roach samples by the predictive model, remains the only valid interpretation of the results. The unanticipated differences in EAA provisioning in the DF-fed and LQD-fed roaches are most likely due to the impacts of dietary quality on gut microbial load (for gut microbial EAA provisioning) and metabolite fluxes to *Blattabacterium* for EAA biosynthesis as outlined above. Finally, the placement of three DF-fed roach gut filtrate samples (filled boxes) and the DF diet samples (two Ds closest to the fungal classifier) within the fungal decision boundary was also unexpected. Despite limited tissue–tissue, diet–tissue isotope fractionations associated with essential amino acids (Martinez del Rio et al., 2009), it is difficult to confidently explain these placements since the compositions and isotopic signatures of the mammalian bone and meat meals, and the soybean meal that make up the composite crude protein fraction of the DF diet are not known. We attribute the positioning of the DF diet and the three DF-fed gut filtrate samples by the predictive model to the presence of brewer’s yeast in the DF diet. Brewer’s yeast is known to be a source of protein in dog foods in general (Martins et al., 2014), and is listed as a component of the crude protein fraction of the dog food used in this study. It is likely that, the use of bacteria and fungi as classifiers and the presence of fungal proteins in the DF diet most likely resulted in the positioning of these samples with the fungal group. Ultimately, the use of a completely different/alternate diet of known composition and comparable protein content to the DF diet (circa 27% protein), and preferably free from bacterial or fungal protein sources, is an appropriate modification to the present experimental set-up, and we recommend this for similar studies.

In conclusion, the determined symbiotic EAA input in both LQD-fed and DF-fed cockroaches in this study highlights the utility of ^13^C-fingerprinting approach in investigating symbiotic function. Dietary quality was shown to influence symbiotic EAA input, although the determined EAA input in DF-fed cockroaches run counter to our expectation of little symbiotic EAA provisioning on a high-quality diet, raising further questions about the roles of the diet, the gut microbiota, the obligate endosymbiont and the host in regulating microbial EAA provisioning, in response to host EAA demands.

## Supplemental Information

10.7717/peerj.2046/supp-1 Table S1 Supplementary Table S1

## Additional Information and Declarations

### Data Availability

The following information was supplied regarding data availability: The raw data has been supplied as Supplemental Information.
