# Supplementary material for: Symbiotic essential amino acids provisioning in the American cockroach, Periplaneta americana (Linnaeus) under various dietary conditions
Source: PeerJ. 2016 May 18;4:e2046. doi: 10.7717/peerj.2046 (PMC4878363; doi:10.7717/peerj.2046)
Supplement: Table S1 [file peerj-04-2046-s001.docx]

**Supplementary Table S1.** Sample sources and mean inter-lab calibrated δ^13^C_EAA_ data used in this study. Means are based on 2 technical replicates for each biological sample and 3 technical replicates for reference samples.

| **ID** | **Sample** | **Sample Type** | **Category** | **LDA** | **Ile** | **Leu** | **Lys** | **Phe** | **Val** |
| --- | --- | --- | --- | --- | --- | --- | --- | --- | --- |
| F1 | Fungi | Ascomycota | Fungi | Training set | -24.0 | -30.6 | -22.6 | -26.9 | -25.6 |
| F2 | Fungi | *Aureobasidium pullulans* | Fungi | Training set | -22.8 | -27.8 | -21.0 | -26.1 | -22.2 |
| F5 | Fungi | unknown | Fungi | Training set | -9.1 | -15.2 | -7.2 | -14.1 | -10.9 |
| F6 | Fungi | unknown | Fungi | Training set | -8.7 | -14.4 | -9.0 | -14.8 | -8.8 |
| F7 | Fungi | unknown | Fungi | Training set | -10.0 | -17.8 | -8.7 | -15.0 | -10.6 |
| F8 | Fungi | *Mortierella alpi* | Fungi | Training set | -6.6 | -13.0 | -5.5 | -12.9 | -9.2 |
| F9 | Fungi | unknown | Fungi | Training set | -9.8 | -17.3 | -7.9 | -14.8 | -12.1 |
| B1 | Bacteria | *Burkholderia xenovorans* | Bacteria | Training set | -12.6 | -13.4 | -4.9 | -18.3 | -14.1 |
| B10 | Bacteria | Bacteria G | Bacteria | Training set | -19.2 | -18.0 | -12.3 | -20.4 | -22.0 |
| B11 | Bacteria | Bacteria H | Bacteria | Training set | -19.1 | -19.6 | -13.1 | -21.5 | -22.7 |
| B12 | Bacteria | Bacteria J | Bacteria | Training set | -24.6 | -24.8 | -19.3 | -28.3 | -26.5 |
| B2 | Bacteria | *Methylobacterium sp.* | Bacteria | Training set | -13.4 | -13.6 | -9.5 | -16.5 | -14.6 |
| B3 | Bacteria | *Klebsiella sp.* | Bacteria | Training set | -19.5 | -20.4 | -14.8 | -24.2 | -21.4 |
| B4 | Bacteria | *Rhodococcus sp.* | Bacteria | Training set | -24.3 | -25.7 | -15.7 | -28.5 | -26.9 |
| B5 | Bacteria | Bacteria B | Bacteria | Training set | -16.6 | -18.3 | -9.3 | -17.9 | -18.3 |
| B6 | Bacteria | Bacteria C | Bacteria | Training set | -17.6 | -19.7 | -9.8 | -19.7 | -21.6 |
| B7 | Bacteria | Bacteria D | Bacteria | Training set | -16.1 | -18.0 | -7.4 | -18.0 | -19.6 |
| B8 | Bacteria | Bacteria E | Bacteria | Training set | -16.6 | -16.4 | -9.0 | -17.3 | -18.2 |
| B9 | Bacteria | Bacteria F | Bacteria | Training set | -21.7 | -24.7 | -16.1 | -26.6 | -25.6 |
| LQC 1 | Cockroach_appendage | LQD roach appendages | LQD roach appendages | Low Quality diet | -20.5 | -25.5 | -14.0 | -24.2 | -22.6 |
| LQC 2 | Cockroach_appendage | LQD roach appendages | LQD roach appendages | Low Quality diet | -21.8 | -26.2 | -15.7 | -24.9 | -23.8 |
| LQC 3 | Cockroach_appendage | LQD roach appendages | LQD roach appendages | Low Quality diet | -20.9 | -25.6 | -12.8 | -24.1 | -22.6 |
| LQG 1 | Cockroach_gut | LQD roach gut filtrate | LQD roach gut filtrate | Low Quality diet | -24.2 | -29.4 | -12.6 | -26.6 | -24.7 |
| LQG 2 | Cockroach_gut | LQD roach gut filtrate | LQD roach gut filtrate | Low Quality diet | -26.3 | -31.4 | -21.3 | -29.7 | -28.2 |
| LQG 3 | Cockroach_gut | LQD roach gut filtrate | LQD roach gut filtrate | Low Quality diet | -23.5 | -28.2 | -17.3 | -25.6 | -24.6 |
| LQD 1 | Diet | Diet | Diet | Training set | -28.3 | -34.0 | -25.6 | -31.7 | -32.2 |
| LQD 2 | Diet | Diet | Diet | Training set | -27.4 | -33.0 | -23.8 | -30.6 | -31.0 |
| LQD 3 | Diet | Diet | Diet | Training set | -27.2 | -33.0 | -22.7 | -30.8 | -31.3 |
| DFC 1 | Cockroach_appendage | DF roach appendages | DF roach appendages | Dog food | -20.8 | -24.3 | -15.2 | -24.1 | -21.7 |
| DFC 2 | Cockroach_appendage | DF roach appendages | DF roach appendages | Dog food | -20.7 | -24.2 | -14.2 | -23.6 | -21.1 |
| DFC 3 | Cockroach_appendage | DF roach appendages | DF roach appendages | Dog food | -20.9 | -24.1 | -13.8 | -23.8 | -21.3 |
| DFC 4 | Cockroach_appendage | DF roach appendages | DF roach appendages | Dog food | -21.4 | -24.6 | -14.9 | -24.3 | -21.8 |
| DFG 1 | Cockroach_gut | DF roach gut filtrate | DF roach gut filtrate | Dog food | -21.2 | -25.4 | -16.6 | -24.5 | -21.7 |
| DFG 2 | Cockroach_gut | DF roach gut filtrate | DF roach gut filtrate | Dog food | -20.0 | -24.9 | -15.7 | -24.2 | -20.8 |
| DFG 3 | Cockroach_gut | DF roach gut filtrate | DF roach gut filtrate | Dog food | -19.7 | -24.5 | -13.5 | -24.4 | -20.4 |
| DFG 4 | Cockroach_gut | DF roach gut filtrate | DF roach gut filtrate | Dog food | -19.9 | -24.1 | -12.9 | -24.3 | -20.0 |
| Dog Food 2 | Diet | Diet | Diet | Training set | -20.5 | -25.8 | -19.2 | -23.1 | -23.1 |
| Dog Food 3 | Diet | Diet | Diet | Training set | -20.6 | -26.3 | -18.0 | -23.3 | -23.5 |
| ALB-FS-1 | *Fusarium spp.* | Test fungus | Test fungus | Test fungus | -20.7 | -27.8 | -19.5 | -26.5 | -22.5 |
| ALB-FS-2-B | *Fusarium spp.* | Test fungus | Test fungus | Test fungus | -23.2 | -29.1 | -23.4 | -27.5 | -25.3 |
